# Dental Practitioners’ Skill Confidence, Arousal, and Filling Quality Under Time Pressure: Analyses of Objective Quality and Subjective–Objective Agreement

**DOI:** 10.3390/dj14100629

**Published:** 2026-10-02

**Authors:** Kaisa M. Leinonen, Jukka Leinonen, Aisha Banafa, Mohammed Al-Haroni, Jan-Are K. Johnsen

**Affiliations:** 1 Department of Clinical Dentistry, Faculty of Health Sciences, UiT The Arctic University of Norway, Hansine Hansens veg 18, 9019 Tromsø, Norway; mohammed.al-haroni@uit.no (M.A.-H.); jan.a.johnsen@uit.no (J.-A.K.J.); 2 Institute of Dentistry, School of Medicine, University of Eastern Finland, Yliopistonranta 1 C, 70210 Kuopio, Finland; jukka.leinonen@uef.fi (J.L.); aisha.banafa@uef.fi (A.B.); 3 Institute of Oral Biology, Faculty of Dentistry, University of Oslo, 0372 Oslo, Norway

**Keywords:** clinical competence, occupational stress, self-assessment, composite resins

## Abstract

**Objectives:** To examine whether domain-specific confidence is associated with filling quality and self-reported arousal under time pressure, and whether self-assessed and externally assessed filling quality agree. **Methods:** In a randomised controlled laboratory experiment, 42 dentists and dental students were assigned to use either the conventional incremental technique or the bulk-fill base technique to fill three cavities. Participants completed practice, timing, and time pressure trials. The baseline skill confidence of the participants was assessed and categorised as high or low. Self-assessed filling quality and self-reported arousal were assessed, and filling quality was also evaluated externally. **Results:** Under time pressure, there was no significant difference in the overall filling quality, incorporating occlusal underfilling and approximal surface quality, between the bulk-fill base and the conventional incremental techniques; however, time pressure increased the proportion of teeth with ≥1 mm occlusal underfilling. Dentists reported higher skill confidence than students for both techniques. The only significant difference in externally assessed filling quality between high- and low-confidence participants occurred under time pressure with the conventional technique: low-confidence participants had more fillings with good quality than high-confidence participants. Regardless of confidence level, arousal increased significantly under time pressure, especially within the conventional incremental group. Low-skill-confidence participants reported higher overall arousal than those with high-skill confidence. Self-assessed and externally assessed quality of the fillings were not associated. **Conclusions:** Neither skill confidence nor self-assessed filling quality reliably reflected externally assessed filling quality. Although neither skill confidence nor the use of the bulk-fill base technique affected quality, both appeared to reduce time-pressure-related arousal.

## 1. Introduction

Health care professionals, including dentists, routinely work in complex, high-pressure environments where emotional states and stress can compromise clinical decision-making and performance [1,2,3,4]. Thus, understanding the psychological factors that influence clinical outcomes is critical for improving the quality of care and supporting professional development [5,6]. One putative psychological factor that may affect clinical performance and outcomes in dentistry is the confidence dental practitioners have in their own abilities. Confidence has already been identified as a relevant issue of dentistry; for example, self-rated confidence was associated with diagnostic accuracy in oral cancer recognition among dentists and dental students [7].

Confidence can be defined as broad, general self-confidence or as domain-specific confidence, which refers to beliefs about one’s competence in performing specialised tasks such as clinical procedures [8,9]. Confidence, especially when aligned with actual skill level, can enhance motivation, decision-making, and clinical accuracy [10,11]. However, confidence does not always correlate with actual competence, and the accuracy of self-assessed performance may vary depending on the type of task [12]. This is also the case among dentists and dental students [13,14]. For example, people may overestimate their capabilities in unfamiliar or high-stress situations [15].

The distinction between confidence and competence remains a recurring challenge in healthcare education and practice [16]. Medical students’ self-assessments were poorly aligned with actual performance, even in a well-defined setting with repeated feedback [17]. Importantly, confidence is not only a cognitive judgement but also involves affective and physiological components, including arousal [18]. Variations in arousal have well-established links to performance, with classic models such as the Yerkes–Dodson inverted-U hypothesis suggesting that both very low and very high arousal can hinder performance, while moderate arousal is typically optimal [19,20]. Considering arousal alongside confidence therefore provides a more comprehensive understanding of factors that may influence clinical performance.

Filling procedures constitute a substantial part of everyday dental practice, and the replacement of existing fillings remains common [21,22]. There is inconsistent evidence explaining restoration failures, yet low immediate quality is generally considered to predispose to secondary caries, fractures and lost restorations [23,24,25]. The quality of resin composite restorations may be especially vulnerable to variations in the operator’s confidence in time pressure settings and when using difficult techniques—especially when many of these characteristics exist simultaneously. Emerging evidence indicates that dental students’ perceptions of procedural difficulty, ease of use, and instrument flexibility may vary considerably across different instruments used in simulated training [26], suggesting that procedural characteristics can influence subjective operator perceptions and confidence during operative tasks.

To our knowledge, there is a lack of studies examining whether clinicians’ confidence in their filling skills is associated with either the quality of the filling or the clinicians’ subjective experiences of performing filling therapy under time pressure. Thus, we set out to investigate the association between confidence in filling therapy and the quality of fillings with or without time-pressure and using two filling techniques. We also investigated arousal levels among subgroups of participants. In addition, we investigated whether participants’ self-assessment of filling quality aligned with external assessment. Specifically, we set out to explore the following: (1) the confidence of dental students and dentists in the conventional incremental and the bulk-fill base techniques, (2) the overall quality of fillings placed with and without time pressure, defined by combining occlusal underfilling and approximal surface quality, (3) the association between confidence and filling quality, (4) the association between self-assessed and externally assessed filling quality, and (5) the association between confidence and arousal during the filling procedure.

## 2. Materials and Methods

### 2.1. Study Design

The present study is based on the same experimental dataset as our previous randomized controlled in vitro trial [27] and the subsequent analyses of arousal and filling quality [3]. Whereas [27] focused on procedure time and approximal filling quality, and [3] examined the effects of time pressure on arousal and filling quality across the two techniques, the present study extends this work by (i) introducing an occlusal underfilling evaluation combined with approximal quality into a novel composite outcome of overall filling quality, (ii) analysing the association between baseline skill confidence and filling quality and arousal, and (iii) examining the agreement between self-assessed and externally assessed filling quality. None of these analyses have been reported previously.

The experiment was carried out in a laboratory dental office; a detailed description of the laboratory setup, cavity dimensions, filling materials, placement protocol, and approximal quality assessment is provided in [27]. In brief, participants performed a standardized Class II restoration on identical maxillary left first molar typodont teeth (DRSK Group AB, Hässleholm, Sweden) mounted in a study model jaw (KaVo, Biberach, Germany) attached to a dental manikin. Each cavity measured 4 mm mesiodistally and 6 mm occlusogingivally; the buccopalatal dimension tapered from approximately 5 mm at the proximal box to 4 mm centrally, with slightly rounded internal angles. Pre-contoured metal matrices (Orbis, Plandent, Helsinki, Finland) with wooden wedges were used. Bonding was performed with Scotchbond™ Universal Plus (3M, Neuss, Germany). Participants in the conventional incremental group used six oblique increments (≤2 mm) of Ceram.x Spectra ST Universal Composite (shade A1; Dentsply Sirona, Konstanz, Germany), each polymerized for 10 s. Participants in the bulk-fill base group used a single 4 mm layer of SDR^®^ flow+ Bulk Fill Flowable (universal shade; Dentsply Sirona) polymerized for 20 s, capped with one horizontal layer of Ceram.x Spectra ST (shade A1), polymerized for 10 s. An LED light-curing unit (Bluephase Style, Ivoclar Vivadent, 1100 mW/cm^2^) was used throughout. Finishing and polishing were not performed. A photograph of the laboratory and the experimental setup is provided in Supplementary Figure S1.

Participants were randomly allocated to either the conventional incremental technique group or the bulk-fill base technique group. The experiment consisted of three trials for both groups. During the first trial, every participant performed a practice filling to get accustomed to the study environment. In the second trial, the time required to fill the cavity was measured in seconds using a handheld stopwatch. For the third trial, participants were requested to fill the cavity under time pressure (Table 1).

The time pressure protocol has been described previously [3]. In brief, technique-specific time limits derived from pilot data were used to scale each participant’s timing-trial duration, corresponding to 68% for the conventional incremental technique and 78% for the bulk-fill base technique. In the timing trial, participants were not given a predetermined time limit; they were allowed to use the time needed to complete the filling, and the time used was recorded. In the time pressure trial, the available time was calculated individually based on each participant’s own timing-trial duration. A visible countdown timer indicated the remaining time, and the procedure was stopped when the timer reached zero.

Participants provided responses regarding perceived skills and filling quality using specific questionnaires (Q1, Q2, Q3, Q4) that featured visual analogue scales (VASs) represented by a 10 cm line. The questionnaires were administered at predefined time points before and after the trial sequence. For pre-trial skill assessment, the endpoints of the VAS were 0 = insufficient and 10 = good. For post-trial filling quality assessment, the endpoints of the VAS were 0 = must be revised and 10 = perfect. The complete set of questionnaires (Q1–Q4) administered before and after each trial is provided in Supplementary Material File S1.

Before the practice trial, the participants completed Q1 to assess their baseline skill confidence in both the conventional incremental technique and the bulk-fill base technique. Regardless of the group to which they were allocated to, each participant assessed their confidence in both techniques using the VAS. The overall mean of these baseline confidence scores was then calculated, and participants were classified as having either “high” or “low” baseline skill confidence based on the mean confidence across the two techniques. Because each participant performed only one technique in the experimental phase, averaging the baseline confidence scores across both techniques provided a general measure of domain-specific confidence for Class II fillings independent of the technique subsequently assigned to the participant.

Participants self-assessed the quality of the filling they placed during the timing and time pressure trials using the 10 cm VAS scale immediately after completing the filling. For the statistical analysis, the continuous VAS scores were divided into three categories to facilitate comparison with the objective assessments: poor quality (0.0–3.3 cm), sufficient quality (3.4–6.6 cm), and good quality (6.7–10.0 cm). The three-band categorisation of the VAS scores (poor: 0.0–3.3 cm; sufficient: 3.4–6.6 cm; good: 6.7–10.0 cm) corresponds to equal thirds of the 10 cm VAS scale, chosen to enable direct comparison with the three-level external evaluation. This categorization allowed the creation of a three-level ordinal variable reflecting participants’ self-estimated quality, which was then cross-tabulated with the objective quality ratings for chi-square analysis.

Self-reported arousal was assessed after each trial using the same VAS-based Short Adjective Check List (SACL) procedure as previously reported [3]. The SACL included two items indicating arousal where the VAS-scale extremes (0 or 10) corresponded to the adjective pairs tense–relaxed and nervous–calm, respectively [28]. Arousal was analysed as the recoded mean of the two SACL items, with higher scores indicating greater arousal.

Approximal surface quality of the fillings was evaluated using modified FDI criteria as described previously [26]. Briefly, marginal gaps under 0.15 mm in diameter or depth were classified as good; gaps between 0.15 mm and 0.25 mm were classified as sufficient/satisfactory; and gaps over 0.25 mm were classified as non-acceptable. Surface porosity was assessed using the same size limits and classification.

Occlusal surfaces of the fillings, including marginal ridges, and comprising the uppermost 2 mm of the restoration, were evaluated specifically for underfilling. Overfilled areas were not evaluated because participants were instructed to only fill the cavity and were not allowed to finish or polish the filling. In a clinical situation, any excess material would typically be removed during finishing and polishing. An occlusal surface was classified as good when its outline and contour were not underfilled. Underfilled areas were evaluated using magnifying loupes, adequate illumination, and a periodontal probe bearing a reference mark at 1 mm from the tip and further classified as either underfilled < 1 mm or underfilled ≥ 1 mm, according to their measured depth.

We created these evaluation criteria for filling quality because the established evaluation systems, such as USPHS and FDI criteria [29,30], were not directly applicable to the present study for the evaluation of occlusal surfaces of fillings. FDI and USPHS criteria assess anatomy primarily as a measure of material wear during clinical service rather than of cavity underfilling. Moreover, the USPHS and FDI criteria do not provide a quantitative method to measure the depth of underfilling.

The evaluations of occlusal surfaces were performed by the first and second authors, both of whom are specialist dentists in cariology and endodontics. The evaluators familiarized themselves with the use of the evaluation protocol in a training session. Both evaluators were blinded to the filling technique used (conventional incremental technique or bulk-fill base technique) and to the time pressure condition (with or without time pressure). The evaluators performed the measurements independently but in the same order. In cases of disagreement, consensus was reached after discussion. Before the consensus discussions, inter-rater reliability for the ordinal occlusal underfilling classification (good, underfilled < 1 mm, underfilled ≥ 1 mm) was calculated using quadratic weighted Cohen’s kappa (κw).

The quality of the whole filling (overall filling quality) was determined by combining the occlusal and approximal quality evaluations. For each filling, the overall quality was assigned according to the lower of the two surface ratings and classified into three categories: good, sufficient, and insufficient.

### 2.2. Ethics

The study was approved by the Norwegian Centre for Research Data (reference number 399613), and the Regional Committees for Medical and Health Research Ethics determined that formal health-research approval was not required (reference number 158600). The experiment was not prospectively registered in a clinical trial registry.

### 2.3. Statistical Analysis

Descriptive statistics (means, standard deviations, frequencies, and percentages) were calculated for demographic variables and questionnaire responses. Inter-rater reliability between the two blinded evaluators for the occlusal underfilling classification was also calculated (quadratic weighted Cohen’s κ = 0.85, 95% CI 0.76–0.93). A paired-samples *t*-test was used to compare confidence assessments between the conventional incremental and the bulk-fill base techniques, while an independent-samples two-sided *t*-test and a chi-square test were used to compare confidence levels between dentists and dental students.

A repeated-measures ANOVA, with trial condition (practice, timing, time pressure) as the within-subject factor and skill confidence (high vs. low) and filling technique (conventional incremental vs. bulk-fill base) as between-subject factors, was used to examine differences in post-performance self-assessments and arousal levels. Bonferroni corrections were applied for post hoc comparisons, and effect sizes were reported as partial eta squared (partial η^2^) for ANOVA analyses, Cohen’s d for *t*-tests, and Cramér’s V for chi-square tests of independence.

Chi-square tests were used to examine associations between filling technique, skill- confidence level, and externally assessed filling quality under time pressure or without time pressure. Associations between self-assessed and externally assessed filling quality were also analysed using chi-square tests.

Beyond the pre-specified primary comparisons, additional subgroup analyses were exploratory in nature. Results from these subgroup analyses should be interpreted with caution due to the increased risk of type-I error, and no adjustment for multiple comparisons was applied to these exploratory tests.

Box plots were used to visually illustrate discrepancies between perceived and actual filling quality. All analyses were conducted using IBM SPSS Statistics 29 and JASP 0.19.3, with statistical significance set at *p* < 0.05.

## 3. Results

The final study sample included 42 participants, comprising 18 graduated dentists and 24 dental students. Women represented 83.3% of the dentists and 75.0% of the students.

### 3.1. The Association Between Filling Technique and Baseline Skill Confidence

Mean baseline confidence for the conventional incremental technique was 7.1 (SD = 2.0) and for the bulk-fill base technique, it was 7.5 (SD = 1.6, *p* = 0.25), t (41) = −1.2, *p* = 0.25, 95% CI [−1.0, 0.3], Cohen’s d = −0.2. The overall mean baseline skill-confidence across techniques was 7.3 (SD = 1.5). For the conventional incremental technique, dentists had higher mean skill-confidence than students (M = 8.5; SD = 1.2 and M = 6.1; SD = 1.8, respectively; t (39) = 5.3, *p* < 0.001, mean difference = 2.4, 95% CI [1.5, 3.4], Cohen’s d = 1.6). Similarly, for the bulk-fill base technique, dentists had higher mean skill-confidence than students (M = 8.2; SD = 1.6 and M = 7.0; SD = 1.5, respectively; t (40) = 2.5, *p* = 0.02, mean difference = 1.2, 95% CI [0.2, 2.2], Cohen’s d = 0.8). When confidence scores were dichotomized based on this mean, 14 dentists and 6 students were categorized as having high-skill confidence, while 4 dentists and 18 students were included in the low-skill-confidence group. Both filling technique groups had 11 low-skill-confidence participants and 10 high-skill-confidence participants.

### 3.2. The Association Between Time Pressure and the Quality of Filling

Across both filling techniques, the proportion of underfilled occlusal surfaces was higher under time pressure compared to the timing trial (X^2^ (df = 4, *n* = 84) = 13.6, *p* = 0.009, Cramér’s V = 0.4; Figure 1). In the timing trial, 17 surfaces were correctly filled, 21 were underfilled by <1 mm, and 4 were underfilled by ≥1 mm; under time pressure, the corresponding numbers were 17, 16, and 9, respectively. In contrast, no significant differences were found when comparing the bulk-fill base and the conventional incremental techniques regarding occlusal underfilling without time pressure (X^2^ (df = 2, *n* = 42) = 1.5, *p* = 0.48, Cramér’s V = 0.2) or with time pressure (X^2^ (df = 2, *n* = 42) = 2.7, *p* = 0.3, Cramér’s V = 0.3).

The overall filling quality was similar for the fillings performed in the timing and time-pressure trials (X^2^ (df = 2, *n* = 42) = 9.2, *p* = 0.56, Cramér’s V = 0.3), and no further differences were observed when looking at the specific filling techniques in timing or time pressure conditions.

### 3.3. The Association Between Baseline Skill Confidence and Occlusal Underfilling

Skill confidence level of the operator was not associated with occlusal underfilling in the timing trial (X^2^ (df = 2, *n* = 42) = 0.5, *p* = 0.76, Cramér’s V = 0.1) or time pressure trial (X^2^ (df = 2, *n* = 42) = 3.9, *p* = 0.14, Cramér’s V = 0.3).

### 3.4. The Association Between Baseline Skill Confidence and Overall Filling Quality

In the timing trial, no significant differences were found in overall filling quality between low-skill-confidence and high-skill-confidence participants across filling techniques (X^2^(df = 2, *n* = 42) = 0.4, *p* = 0.84, Cramér’s V = 0.1) or when analysed separately by filling technique (conventional incremental technique: X^2^ (df = 2, *n* = 21) = 0.2, *p* = 0.93, Cramér’s V = 0.09; bulk-fill base technique: X^2^ (df = 2, *n* = 21) = 1.3, *p* = 0.53, Cramér’s V = 0.3).

In the time pressure trial, 46% of fillings performed by low-skill-confidence participants were evaluated as good in overall quality, whereas 15% of fillings performed by high-skill-confidence participants were evaluated as good in overall quality; however, this difference was not statistically significant (X^2^ (df = 2, *n* = 42) = 4.6, *p* = 0.10, Cramér’s V = 0.3). Of the nine fillings evaluated as insufficient, five were placed by high-skill-confidence participants and four by low-skill-confidence participants. With the conventional incremental technique under time pressure none (0%) of the fillings placed by participants with high-skill-confidence were evaluated as good, compared to 36% of fillings placed by the low-skill-confidence participants (X^2^ (df = 2, *n* = 21) = 6.9, *p* = 0.03, Cramér’s V = 0.6).

### 3.5. The Associations Between Arousal, Baseline Skill Confidence and Time Pressure

Participants with low-skill-confidence reported higher overall arousal than those with high- skill-confidence across all trials (Figure 2; F (1, 40) = 4.3, *p* = 0.046, partial η^2^ = 0.10).

For both skill confidence groups, the self-reported arousal levels were higher in the time pressure trial compared to the practice and timing trials (Figure 3; F (2, 80) = 15.4, *p* < 0.001, partial η^2^ = 0.28). Post hoc Bonferroni-adjusted comparisons confirmed that arousal during the time pressure trial was significantly higher than that in the practice and timing trials (*p* = 0.005 and *p* = 0.019, respectively).

In the conventional incremental group, arousal did not differ significantly between high- and low-skill-confidence participants (F (1,19) = 0.6, *p* = 0.44, partial η^2^ = 0.03, Figure 4). Even so, in the conventional incremental group, arousal increased significantly from the practice trial to the timing trial but reached the highest level under time pressure for both skill confidence groups (F (2, 38) = 20.1, *p* < 0.001, partial η^2^ = 0.51). For high-skill-confidence participants, arousal during the time pressure trial was significantly greater than during both the practice (*p* = 0.015) and timing trials (*p* = 0.008).

In the bulk-fill base group, low-skill-confidence participants reported higher overall arousal than high-skill-confidence participants (F (1, 19) = 4.6, *p* = 0.045, partial η^2^ = 0.20, Figure 3). However, the differences in arousal between trials were not statistically significant (F (2, 38) = 1.4, *p* = 0.261, partial η^2^ = 0.068).

### 3.6. The Association Between Subjective and Objective Filling Quality

There was no significant association between self-assessed and externally assessed quality of fillings (X^2^ (df = 4, *n* = 82) = 5.5, *p* = 0.24, Cramér’s V = 0.2; Table 2, Figure 4). Two participants did not self-assess the quality of their fillings after the time pressure trial.

## 4. Discussion

To the best of our knowledge, few studies have simultaneously examined the associations between skill confidence, clinical performance, and arousal under time pressure. The principal findings were twofold. First, although graduated dentists possessed higher skill confidence, this did not reliably predict filling quality. Under time pressure, higher skill confidence was in fact inversely associated with filling quality, but only when using the conventional incremental technique. Second, self-assessed filling quality was not aligned with externally assessed filling quality.

### 4.1. The Association Between Filling Technique and Baseline Skill Confidence

Consistent with previous findings, dentists were more confident than dental students, which may reflect greater clinical experience and more developed domain-specific self-efficacy [7,31,32]. Through repeated exposure to varied clinical procedures, practising dentists typically develop stronger self-efficacy, whereas students, still refining their skills, tend to report lower confidence [31,33]. Notably, the confidence difference between dentists and students was more pronounced for the conventional incremental technique than for the bulk-fill base technique. Evidence from medical practice indicates that procedural confidence is positively associated with the number of times a procedure has been performed, with recent procedural experience emerging as the strongest predictor [34].

An important consideration is that the low- and high-confidence groups were confounded with dentist/student status: the low-confidence group was predominantly composed of dental students (18 of 22, 82%), while the high-confidence group was predominantly composed of practicing dentists (14 of 20, 70%). Consequently, the subgroup finding that low-confidence participants produced more high-quality fillings under time pressure in the conventional incremental technique may equally reflect student status rather than lower confidence per se. This interpretation is consistent with our previous work on the same dataset, which reported that students produced more high-quality fillings than dentists using the bulk-fill base technique under time pressure [3]. Whether the observed pattern reflects confidence level, dentist/student status, or their interaction cannot be disentangled with the present sample.

### 4.2. The Association Between Time Pressure and the Quality of Fillings

In the present study, no significant differences were observed in the overall filling quality, defined by combining occlusal underfilling and approximal surface quality, between the conventional incremental and the bulk-fill base techniques, neither with nor without time pressure. However, under time pressure, the number of occlusal surfaces underfilled by ≥1 mm was higher than in the timing trial. Time pressure can impair concentration and fine motor control, particularly during tasks that require sustained attention and precise execution [35,36,37]. These factors may have contributed to the observed increase in the number of fillings with underfilled occlusal surfaces.

### 4.3. The Association Between Baseline Skill Confidence and Filling Quality

When both filling techniques were considered together, the difference in overall filling quality between low- and high-confidence participants under time pressure was not statistically significant. However, when analysed separately by technique, higher skill confidence was associated with fewer high-quality fillings in the time pressure trial in the conventional incremental technique group, but not in the bulk-fill base technique group and not in the timing trial. The finding aligns with broader evidence that healthcare professionals’ subjective confidence is often poorly calibrated to actual performance [38]. Among nurses, higher confidence was not associated with better judgement accuracy, and time pressure showed no statistically significant effect on accuracy [9]. However, time pressure interacts with task difficulty by increasing confidence in easy judgements and decreasing confidence in difficult judgements but without improving judgement accuracy [9]. Among dentists, higher confidence does not correspond to more appropriate antibiotic prescribing decisions [39]. Also, dentists’ self-estimated competence in managing dental trauma does not reflect actual knowledge level [13]. In contrast, higher confidence was associated with greater diagnostic accuracy overall, although this association weakened in more difficult clinical cases [7]. The mechanism underlying our finding may relate to time pressure impairing executive control [36]. This may be due to increased mental workload and reduced functioning of brain systems involved in executive control [35].

The filling quality results presented in this article build on our previously published work [26] extending the evaluation from approximal surface quality to the quality of the entire filling, including occlusal surfaces. As occlusal surfaces were previously excluded, we have now developed a method to incorporate them, enabling new insights into comprehensive filling quality. The conventional incremental technique is more technique sensitive and time-consuming than the bulk-fill base technique [40,41]. The combination of time pressure and the conventional incremental technique seems to have compromised filling quality, particularly among high-skill-confidence participants, as the difference in filling quality between high- and low-skill-confidence groups occurred only under these conditions. In this subgroup, one possible interpretation is that a degree of self-doubt may enhance performance, as suggested by previous research on defensive pessimism [42,43]. However, our study cannot confirm this mechanism, and this hypothesis warrants further investigation. An alternative explanation is that difficult tasks may lead some participants to evaluate their abilities more conservatively, which could partly explain why lower reported skill confidence was observed alongside adequate performances [44]. Both interpretations remain speculative and require further investigation.

### 4.4. The Associations Between Arousal, Baseline Skill Confidence and Time Pressure

Regardless of the confidence level, arousal increased under time pressure but only when using the conventional incremental technique. This suggests that time pressure may be particularly stress-inducing when the task involves a more technique-sensitive filling procedure. Although the low-confidence participants reported higher arousal, the quality of their fillings was similar to or better than that of the high-confidence participants. This aligns with previous findings that moderate arousal can enhance performance by sharpening focus and task engagement [45,46]. When using the bulk-fill base technique, high-confidence participants consistently reported lower arousal than their low-confidence peers. The bulk-fill base technique’s procedural simplicity combined with confidence may have helped to reduce arousal brought on by time pressure. A comparable pattern was observed among dental professionals exhibiting overconfidence in their mentalisation skills [47]. They reported better quality of life, likely because overconfidence functioned as a buffer against clinically related stress [47]. However, this may have come at the cost of reduced accuracy in perceiving patients’ emotional states [47]. Recent evidence indicates that dental students’ perceptions of ease of use, flexibility, and procedural difficulty may vary considerably according to the instruments used in simulated training, with simpler and more forgiving instruments perceived more favourably [26]. Although operator stress was not directly measured, the authors suggested that such procedural characteristics may contribute to a less demanding learning experience and potentially reduce operator stress during preclinical training [26].

### 4.5. The Association Between Subjective and Objective Filling Quality

We found that the self-assessed and externally assessed filling quality were not associated. Most low-performing participants overestimated the quality of the fillings they had placed, while half of the higher performers underestimated the quality of the fillings they had placed. This pattern resembles previously reported confidence-performance discrepancies but could also be explained by regression to the mean [44,48]. However, the mismatch between self-assessed and externally assessed quality of fillings may not solely reflect poor self-judgement but also represent the influence of task difficulty and testing conditions that make accurate self-assessment challenging [44].

Similar discrepancies between perceived and actual ability have been reported across clinical contexts. For example, dentists overestimate their accuracy in assessing patients’ anxiety [49]. Similarly, among dental and medical students, lower-performing students tend to overestimate their competence, whereas higher-performing students tend to slightly underestimate their competence [50,51,52].

## 5. Limitations

No previously published or pilot data were available at the time of the original experiment to reliably estimate an appropriate a priori sample size for this study [3,27]. The final sample of 42 participants was recruited as a convenience sample based on the feasibility of recruitment among practicing dentists in Troms County (Norway) and 4th- and 5th-year dental students at UiT The Arctic University of Norway. Nevertheless, the modest sample size may have limited statistical power for smaller subgroup effects and should be considered when interpreting non-significant findings. The convenience-sampling approach may also limit the generalizability of the results to broader populations of dental professionals and students.

The sample size may limit the generalizability of these findings to broader populations of dental professionals and students. The predominance of female participants (83.3% among dentists and 75.0% among students) may have influenced the reported confidence and arousal levels, as prior literature suggests possible gender-related differences in self-rated confidence and stress responses in healthcare settings. This should be considered when interpreting the findings and generalising them to more gender-balanced populations. Although the experimental tasks simulated clinical conditions, they were conducted in a controlled laboratory setting rather than actual patient-care environments, potentially reducing ecological validity. Self-reported visual analogue ratings of confidence, arousal, and performance may introduce response bias or miscalibration [53]. Confidence reflects perceived, rather than actual, ability and can be influenced by cognitive or personality factors such as anxiety or self-efficacy. Dichotomisation of the continuous baseline confidence scores at the mean value inevitably resulted in loss of information relative to the original continuous scale. This should be considered when interpreting the subgroup analyses, and a continuous-variable analysis is a natural extension for future research with a larger sample. Participants’ specific prior clinical experience with each of the two filling techniques was not systematically recorded, and dentist/student status was used as a proxy for overall clinical experience. Future studies should collect more detailed information on prior technique-specific experience to enable a more direct analysis of its influence on confidence.

Some of the chi-square analyses comparing filling quality between the timing and time-pressure trials do not fully account for the within-participant correlation inherent in the repeated-measures design. This limitation should be considered when interpreting these results, although the observed effect sizes and consistency across analyses support the robustness of the main findings. Some chi-square subgroup analyses had a small proportion of cells with expected counts below 5, which may reduce the reliability of the chi-square approximation. Results from these subgroup analyses should be interpreted with caution. Finally, while time pressure served as the primary stressor, other clinically relevant stressors, such as interpersonal challenges or decision fatigue, were not examined and warrant investigation in future work.

## 6. Conclusions

Low self-confidence was generally associated with higher arousal levels. Although the overall association between confidence and filling quality under time pressure was not statistically significant when both techniques were considered together, low-confidence participants using the conventional incremental technique produced more high-quality fillings than high-confidence participants under time pressure. This subgroup finding should be interpreted with caution and warrants further investigation. The bulk-fill base technique was associated with less variation in arousal and filling quality under time pressure. These laboratory findings may suggest potential benefits for procedural efficiency, but any implications for stress regulation in clinical practice require further investigation in real-world settings. Incorporating more metacognitive training into dental curricula may help students develop more accurate self-assessment of their clinical abilities as independent practitioners [54].

## Figures and Tables

**Figure 1:**
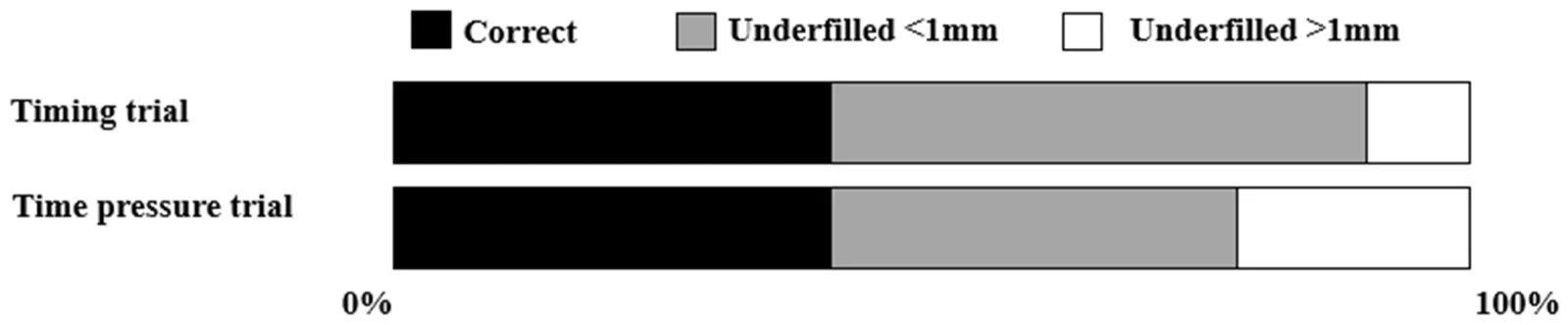
The distribution of occlusal under filling under normal and time-pressure conditions. The frequencies in the normal condition were 17, 21, and 4 (total *n* = 42), and in the time-pressure condition, the frequencies were 17, 16, and 9 (total *n* = 42).

**Figure 2:**
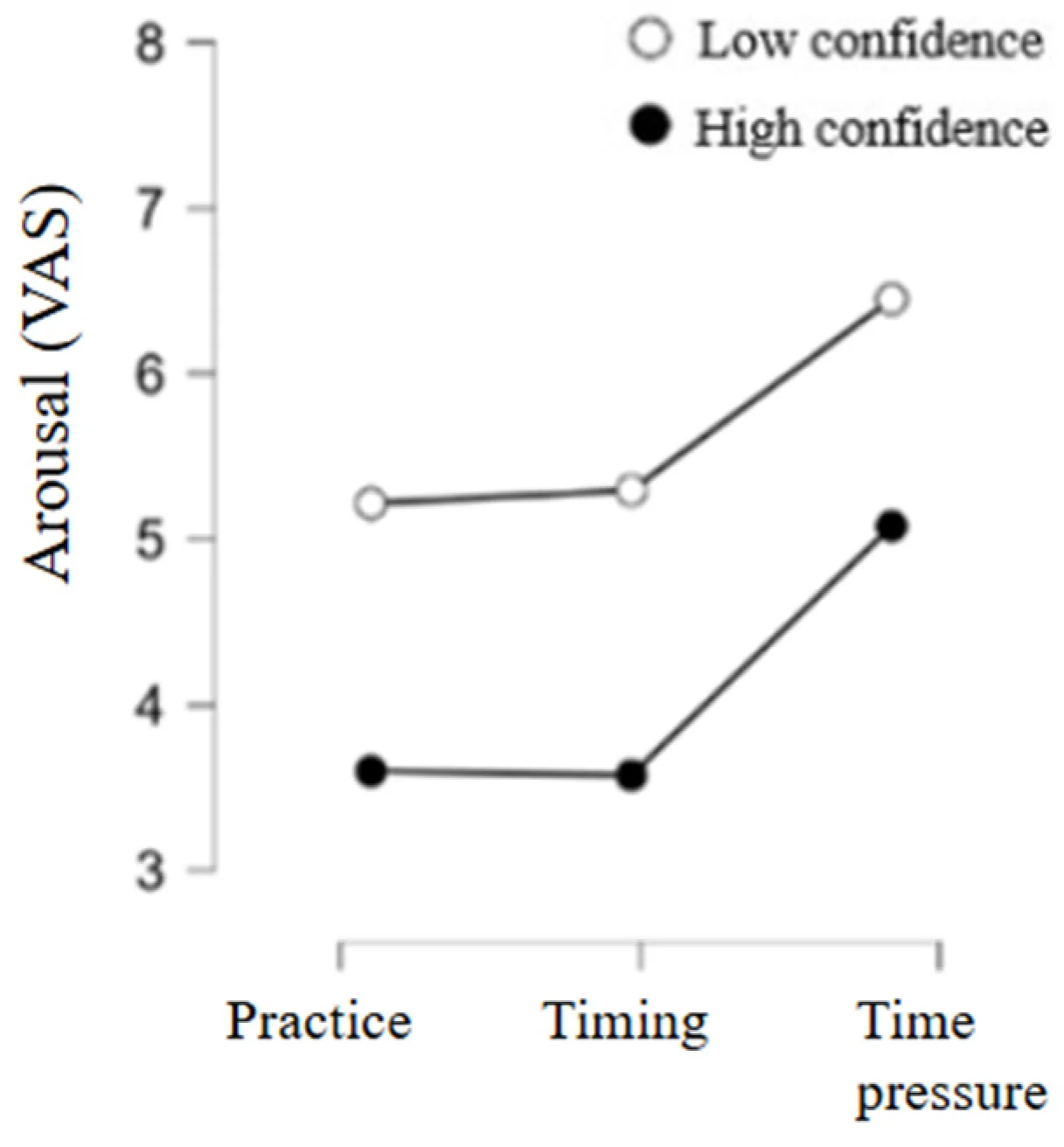
The association of baseline skill confidence on the arousal levels across experimental trials.

**Figure 3:**
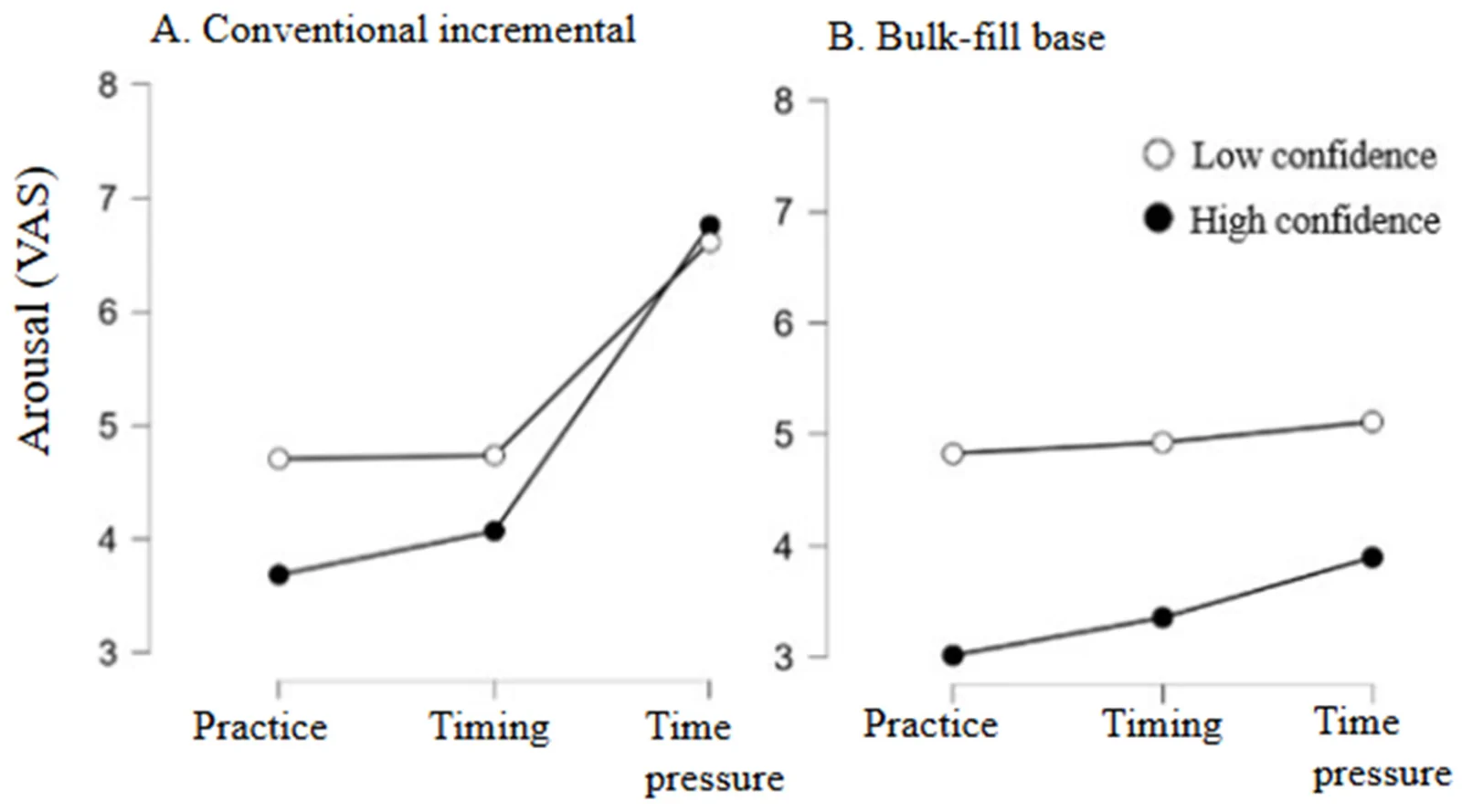
Mean arousal across practice, timing, and time pressure trials for low-skill-confidence and high-skill-confidence participants in the (**A**) conventional incremental group and (**B**) bulk-fill base group.

**Figure 4:**
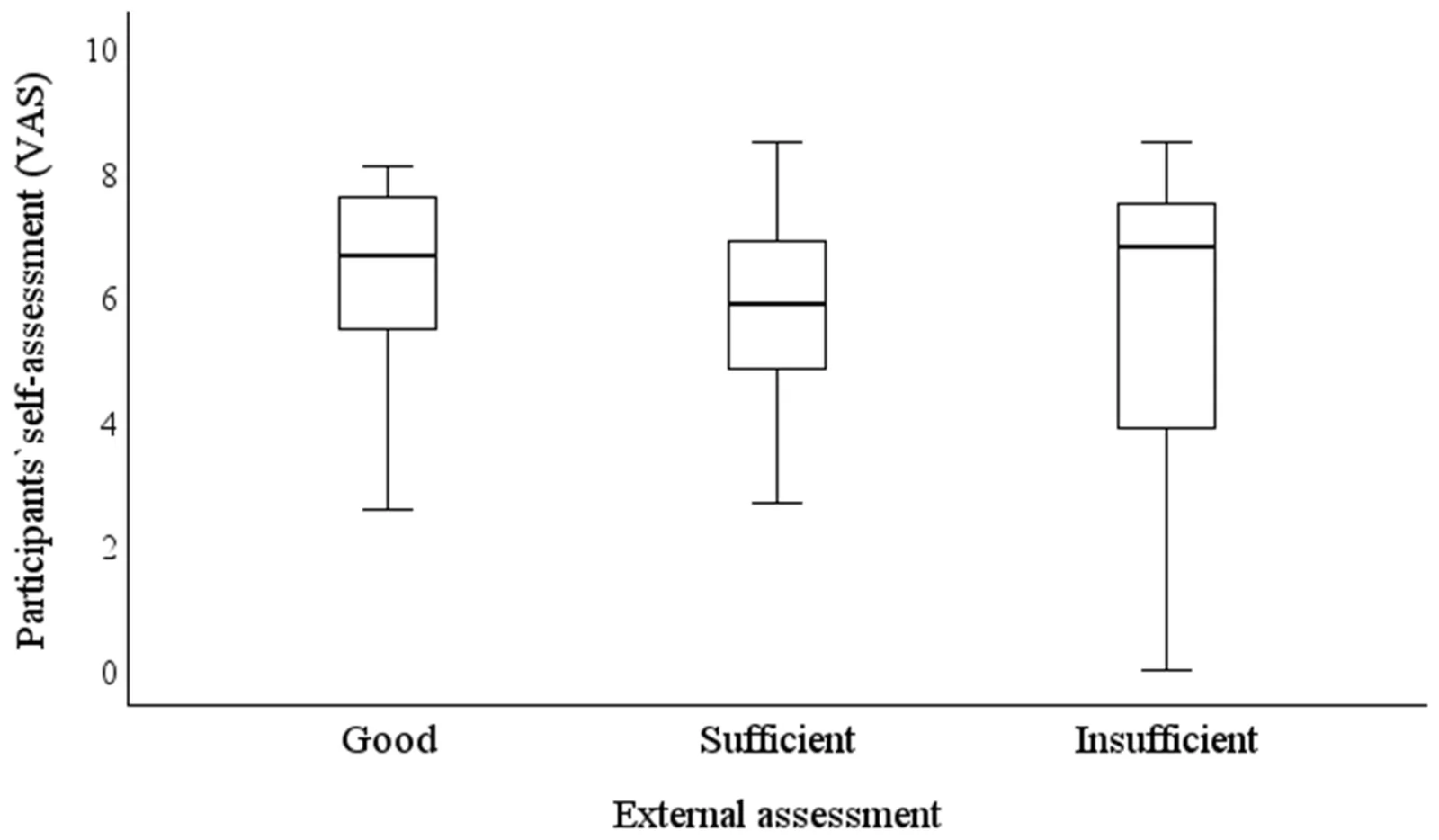
The boxplot illustrates the distribution of participants’ self-assessed quality scores within each external assessment category including the median, interquartile range and overall spread of the data.

**Table 1:** Study design.

Study Phase	Description
Participants	*n* = 42
Group 1	Conventional incremental group (*n* = 21)
Group 2	Bulk-fill base group (*n* = 21)
Trial 1	Practice
Trial 2	Timing
Trial 3	Time pressure
Questionnaires (Q1–Q4)	Completed before and after each trial

**Table 2:** Alignment between external assessment and participants’ self-assessment of the filling quality.

External Assessment	Self-Assessment: Insufficient	Self-Assessment: Sufficient	Self-Assessment: Good	Total
Insufficient	2	4	7	13
Sufficient	3	27	13	43
Good	1	13	12	26
Total	6	44	32	82

## Data Availability

The data is not publicly available because it contains information that could compromise the privacy of research participants.

## Supplementary Materials

The following supporting information can be downloaded at: https://www.mdpi.com/article/10.3390/dj14100629/s1.
