# Supplementary material for: Dental Practitioners’ Skill Confidence, Arousal, and Filling Quality Under Time Pressure: Analyses of Objective Quality and Subjective–Objective Agreement
Source: Dent J (Basel). 2026 Oct 2;14(10):629. doi: 10.3390/dj14100629 (PMC13703584; doi:10.3390/dj14100629)
Supplement: Supplementary file 1 [file dentistry-14-00629-s001.zip › File S1.pdf]

Complete set of questionnaires translated form Norwegian into English; all subgroups will receive the same questionnaire package after randomisation to either the "bulk-fill group" or the "conventional incremental group".

BULK-FILL Group

Q1a: Questionnaire 1a

How would you generally rate your skill in performing fillings using the bulk-fill technique? Place a single mark on the line that corresponds to your assessment, where the endpoints correspond to insufficient skill and good skill, respectively.

insufficient good

I-- I

How would you generally rate your skill in performing fillings using the conventional incremental technique? Place a single mark on the line that corresponds to your assessment, where the endpoints correspond to insufficient skill and good skill, respectively.

insufficient good

I-- I

Q1b: Questionnaire 1b

Gender

- ☐ Female
- ☐ Male
- ☐ Non-binary
- ☐ Prefer not to disclose

Age

- ☐ 20-25
- ☐ 26-30
- ☐ 31-35
- ☐ 36-40
- ☐ 41-45
- ☐ 46-50
- ☐ 51-55
- ☐ 56-60
- ☐ 61-65

Clinical work experience

Student

- ☐ Fourth year
- ☐ Fifth year

Dentist

- ☐ 0-5 years
- ☐ 6-10 years
- ☐ 11-15 years
- ☐ 16-20 years
- ☐ 21-25 years
- ☐ 26-30 years
- ☐ Over 30 years

Which technique do you usually use when filling a 16do cavity?

- ☐ Conventional/traditional technique with oblique layers of conventional composite
- ☐ Flowable "flow" bulk-filling technique (with conventional composite on top)
- ☐ Other, please specify:______________________________

How would you rate your skill right now in performing fillings using the bulk-fill technique? Place a single mark on the line that corresponds to your assessment, where the endpoints correspond to insufficient skill and good skill, respectively.

insufficient good

I--- I

Expected time to complete the filling (in minutes): _____

Expected quality of the filling

Indicate the expected quality of the filling you are about to perform on the line below by placing a single mark. The endpoints represent a perfect filling and a failed filling, respectively. You may place your mark anywhere on the line that corresponds to your expectation of your own performance.

must be revised perfect

I--- I

Self-reported stress levels
Indicate how you feel right now by placing a single mark on the lines below. The endpoints represent experiencing various forms of stress (tension/nervousness) and the absence of stress (relaxed/calm), respectively. You may place your mark anywhere on the lines that corresponds to your experience. Place one mark on each line.

tense relaxed

I--- I

nervous calm

I--- I

Q2a: Questionnaire 2a

Self-assessed quality of the filling

Indicate the estimated quality of the filling you have just performed on the line below by placing a single mark. The endpoints represent a perfect filling and a failed filling, respectively. You may place your mark anywhere on the line that corresponds to your performance.

must be revised perfect

I--- I

Expected quality of the filling

Indicate the expected quality of the filling you are about to perform on the line below by placing a single mark. The endpoints represent a perfect filling and a failed filling, respectively. You may place your mark anywhere on the line that corresponds to your expectation of your own performance.

must be revised perfect

I--- I

Self-reported stress levels

Indicate how you feel right now by placing a single mark on the lines below. The endpoints represent experiencing various forms of stress (tension/nervousness) and the absence of stress (relaxed/calm), respectively. You may place your mark anywhere on the lines that corresponds to your experience. Place one mark on each line.

tense relaxed

I--- I

nervous calm

I--- I

Q2b: Questionnaire 2b

Self-assessed quality of the filling

Indicate the estimated quality of the filling you have just performed on the line below by placing a single mark. The endpoints represent a perfect filling and a failed filling, respectively. You may place your mark anywhere on the line that corresponds to your performance.

must be revised perfect

I--- I

Self-reported stress levels

Indicate how you feel right now by placing a single mark on the lines below. The endpoints represent experiencing various forms of stress (tension/nervousness) and the absence of stress (relaxed/calm), respectively. You may place your mark anywhere on the lines that corresponds to your experience. Place one mark on each line.

tense relaxed

I--- I

nervous calm

I--- I

CONVENTIONAL INCREMENTAL group

Q1a: Questionnaire 1a

How would you generally rate your skill in performing fillings using the bulk-fill technique? Place a single mark on the line that corresponds to your assessment, where the endpoints correspond to insufficient skill and good skill, respectively.

insufficient good

I--- I

How would you generally rate your skill in performing fillings using the conventional incremental technique? Place a single mark on the line that corresponds to your assessment, where the endpoints correspond to insufficient skill and good skill, respectively.

insufficient good

I--- I

Q1b: Questionnaire 1b

Gender

- Female
- Male
- Non-binary
- Prefer not to disclose

Age

- 20-25
- 26-30
- 31-35
- 36-40
- 41-45
- 46-50
- 51-55
- 56-60
- 61-65

Clinical work experience

Student

- Fourth year
- Fifth year

Dentist

- 0-5 years
- 6-10 years
- 11-15 years
- 16-20 years
- 21-25 years
- 26-30 years
- Over 30 years

Which technique do you usually use when filling a 16do cavity?

- Conventional/traditional technique with oblique layers of conventional composite
- Flowable "flow" bulk-filling technique (with conventional composite on top)
- Other, please specify:______________________________

How would you rate your skill right now in performing fillings using the conventional incremental technique? Place a single mark on the line that corresponds to your assessment, where the endpoints correspond to insufficient skill and good skill, respectively.

insufficient good

I--- I

Expected time to complete the filling (in minutes): _____

Expected quality of the filling

Indicate the expected quality of the filling you are about to perform on the line below by placing a single mark. The endpoints represent a perfect filling and a failed filling,

respectively. You may place your mark anywhere on the line that corresponds to your expectation of your own performance.

must be revised perfect

I--- I

Self-reported stress levels

Indicate how you feel right now by placing a single mark on the lines below. The endpoints represent experiencing various forms of stress (tension/nervousness) and the absence of stress (relaxed/calm), respectively. You may place your mark anywhere on the lines that corresponds to your experience. Place one mark on each line.

tense relaxed

I--- I

nervous calm

I--- I

Q2a: Questionnaire 2a

Self-assessed quality of the filling

Indicate the estimated quality of the filling you have just performed on the line below by placing a single mark. The endpoints represent a perfect filling and a failed filling, respectively. You may place your mark anywhere on the line that corresponds to your performance.

must be revised perfect

I--- I

Expected quality of the filling

Indicate the expected quality of the filling you are about to perform on the line below by placing a single mark. The endpoints represent a perfect filling and a failed filling, respectively. You may place your mark anywhere on the line that corresponds to your expectation of your own performance.

must be revised perfect

I--- I

Self-reported stress levels

Indicate how you feel right now by placing a single mark on the lines below. The endpoints represent experiencing various forms of stress (tension/nervousness) and the absence of stress (relaxed/calm), respectively. You may place your mark anywhere on the lines that corresponds to your experience. Place one mark on each line.

tense relaxed

I--- I

nervous calm

I--- I

Q2b: Questionnaire 2b

Self-assessed quality of the filling

Indicate the estimated quality of the filling you have just performed on the line below by placing a single mark. The endpoints represent a perfect filling and a failed filling, respectively. You may place your mark anywhere on the line that corresponds to your performance.

must be revised perfect

I--- I

Self-reported stress levels

Indicate how you feel right now by placing a single mark on the lines below. The endpoints represent experiencing various forms of stress (tension/nervousness) and the absence of stress (relaxed/calm), respectively. You may place your mark anywhere on the lines that corresponds to your experience. Place one mark on each line.

tense relaxed

I--- I

nervous calm

I--- I
